# The Anti-Obesity Effect of Traditional Chinese Medicine on Lipid Metabolism

**DOI:** 10.3389/fphar.2021.696603

**Published:** 2021-06-21

**Authors:** Qijing Fan, Furong Xu, Bin Liang, Xiaoju Zou

**Affiliations:** ^1^ College of Chinese Materia Medica and Yunnan Key Laboratory of Southern Medicinal Utilization, Yunnan University of Chinese Medicine, Kunming, China; ^2^ Center for Life Sciences, School of Life Sciences, Yunnan University, Kunming, China

**Keywords:** traditional Chinese medicines, obesity-related metabolic diseases, anti-obesity effect, lipid metabolism, mechanisms

## Abstract

With the improvement of living conditions and the popularity of unhealthy eating and living habits, obesity is becoming a global epidemic. Obesity is now recognized as a disease that not only increases the risk of metabolic diseases such as type 2 diabetes (T2D), non-alcoholic fatty liver disease (NAFLD), cardiovascular disease (CVD), and cancer but also negatively affects longevity and the quality of life. The traditional Chinese medicines (TCMs) are highly enriched in bioactive compounds and have been used for the treatment of obesity and obesity-related metabolic diseases over a long period of time. In this review, we selected the most commonly used anti-obesity or anti-hyperlipidemia TCMs and, where known, their major bioactive compounds. We then summarized their multi-target molecular mechanisms, specifically focusing on lipid metabolism, including the modulation of lipid absorption, reduction of lipid synthesis, and increase of lipid decomposition and lipid transportation, as well as the regulation of appetite. This review produces a current and comprehensive understanding of integrative and systematic mechanisms for the use of TCMs for anti-obesity. We also advocate taking advantage of TCMs as another therapy for interventions on obesity-related diseases, as well as stressing the fact that more is needed to be done, scientifically, to determine the active compounds and modes of action of the TCMs.

## Introduction

The state of being overweight and obesity are defined as the abnormal or excessive accumulation of fat, mostly in the form of triacylglycerols (TAGs) and cholesterol esters (CEs) in adipose and non-adipose tissues or organs. The World Health Organization (WHO) classification uses body mass index (BMI) to define overweight as being 25–29.9 kg/m^2^ and obesity as being ≥30 kg/m^2^ (https://www.who.int/topics/obesity/zh/). Along with an increasing accessibility to food and the popularity of unhealthy lifestyles, obesity is becoming a global epidemic and its metabolic consequences are currently among the most pressing public health challenges (Hossain et al., 2007). The primary consequence of obesity is associated with an increased mortality and a susceptibility to comorbidities, with few viable therapeutic interventions being available. Today, obesity is increasingly gaining attention due to its intimate association with a growing list of diseases beyond T2D, NAFLD, cancers (Stoll, 1998; Arem and Irwin, 2013; Seo et al., 2015; Incio et al., 2018), and CVD (Lazo and Clark, 2008; Zhao et al., 2019), such as atherosclerosis (AS) (Rocha and Libby, 2009; Aboonabi et al., 2019). Meanwhile, obesity also has a substantial impact on the quality of life. Obesity is usually associated with a lower health-related quality of life than those possessing a normal weight (Pinhas-Hamiel et al., 2006). In children and adolescents, obesity is usually associated with sedentary lifestyles, lower levels of self-esteem, social exclusion, poor educational achievements, and so on (de Beer et al., 2007; Wille et al., 2008). In addition, global health costs associated with obesity and its complications are estimated to be ∼US$2 trillion (Dobbs et al., 2014). If the prevalence of obesity continues on its rising trend, almost half of the world’s adult population could be overweight or obese by 2030, imposing even greater personal, social, and economic costs (https://www.mckinsey.com/industries/healthcare-systems-and-services).

Effectively combating obesity around the world may require a comprehensive strategy involving multiple interventions (Dobbs et al., 2014). Management of obesity is aimed at weight loss, which improves the quality of life. Studies have shown that weight loss after treatment was associated with varying degrees of improvement in obesity-related psychosocial problems, physical functioning, physical role functioning, bodily pain, general health, mental health, and vitality (Kaukua et al., 2003; Pearl et al., 2018). To lose weight, lifestyle interventions, dietary changes, and physical activity are the first-line approaches, followed by medical treatment and bariatric surgery. Thus far, several drugs have been approved for weight loss, such as orlistat, liraglutide, lorcaserin, and diethylpropion (Solas et al., 2016; Gadde et al., 2018). However, in addition to the considerable financial cost of these drugs, numerous side effects have been increasingly reported, such as headache, dizziness, fatigue, nausea, dry mouth, insomnia, anxiety, and constipation (Smith et al., 2010; Aronne et al., 2013; Pi-Sunyer et al., 2015; Nissen et al., 2016; Solas et al., 2016; Gadde et al., 2018).

The pathogenesis of obesity is complex and determined by the interaction of genetic, environmental, and psychosocial factors acting through several physiological mediators (González-Muniesa et al., 2017). Many studies have reported that lipid metabolic pathways are a potential therapeutic target to prevent or delay the occurrence and progression of obesity and obesity-related metabolic diseases, as the major physiological factor in these diseases is the disturbance of lipid metabolism, such as a dysfunction in lipid absorption, lipid synthesis, lipid decomposition, and/or lipid transportation (Meikle and Summers, 2017). Compared with the modern drugs mentioned above, TCMs have been widely used to treat obesity for a very long time. This fact alone suggests that TCMs may be used as a vast resource for the development of natural anti-obesity drugs possessing fewer side effects (Li et al., 2017; Martel et al., 2017; Zhang Y. et al., 2018; Zhang et al., 2020; Ji-Ping et al., 2021). While some of the anti-obesity effects and mechanisms of TCMs have been studied in the past decade, most of these studies only focused on single/several genes or signaling pathways involved in lipid metabolism. Our goal in this review is to collate these data and give a systematic and comprehensive overview of the anti-obesity effects and mechanisms of TCMs and their major ingredients by targeting lipid metabolism.

## The Traditional Chinese Medicines With Anti-Obesity Effects

The diverse evolution of plants represents a near inexhaustible source of biologically active compounds. The importance of these natural products for medicine and health has been immense (Courdavault et al., 2020). For many years, scientists have been researching and applying natural, plant-derived preparations as medicine in clinical treatment. Through these actions, active compounds such as strychnine and brucine (Langley, 1918), quinine (Dickson, 1823), colchicine (1890), caffeine (Bennett, 1873), and artemisinin (Ma et al., 2020) have been discovered. Thus, natural products provide important clues to the identification and development of synergistic drugs.

TCM refers to substances used for the prevention, diagnosis, and treatment of diseases, as well as for rehabilitation and health care under the guidance of TCM theories. It may be the best resource for the use of natural products, and it represents the accumulated experiences of thousands of years of medical practice. The written records of TCM date back at least 2,000 years to *Shen Nong’s Classic of Materia Medica*. In the long history of China, TCM has made an indelible contribution to the health of the Chinese people. Moreover, based on 2,000 years of experience in the use of TCMs and modern scientific research, the eleventh edition of the *Pharmacopoeia of the People’s Republic of China (ChP)* was promulgated and implemented in 2020. In this volume, the *ChP* stipulated the processing, usage, dosage, and compatibility of TCMs and included many classic prescriptions (Chinese Pharmacopoeia Commission).

Notably, in recent decades, there has been a growing interest in TCM. Since 1973, there have been 2,104 articles related to “traditional Chinese medicine” AND “lipid metabolism” in PubMed, while in 2020 alone, there were 389 articles (PubMed, https://pubmed.ncbi.nlm.nih.gov, last accessed on March 31, 2021). And 35 individual prescriptions from the *ChP (2020 edition)* were identified, each of which is clearly indicated as being anti-obesity and anti-hyperlipidemia (Table 1). Based on the formulation of these 35 prescriptions, we searched the Latin name of each single TCM for “anti-obesity” OR “anti-hyperlipidemia” in PubMed and CNKI in recent decades. Five TCMs including *Crataegus pinnatifida* Bunge, *Salvia miltiorrhiza* Bunge, *Polygonum multiflorum* Thunb., *Alisma plantago-aquatica* L., and *Panax notoginseng* (Burkill) F.H. Chen were identified. Each of these plants was implicated not only to function in anti-obesity and anti-hyperlipidemia but also to be mechanistically associated with these processes. Also, based on the widespread use of edible TCMs among people, some medicinal plants appear in diets. We also reviewed commonly used edible TCMs possessing the effects of anti-obesity and anti-hyperlipidemia including *Scutellaria baicalensis* Georgi, *Curcuma longa* L., pu-erh tea, green tea, *Tripterygium wilfordii* Hook. f., chilli peppers, and grape and the main bioactive compounds from them, such as baicalin, curcumin, epigallocatechin gallate, green tea polyphenol, triptolide and celastrol, capsaicin, and resveratrol (Sham et al., 2014; Wang S., et al., 2014; Martel et al., 2017).

**TABLE 1:** TCM anti-obesity formulations from the *Pharmacopoeia of the People’s Republic of China (ChP) (2020 edition)*.

TCM formulation of anti-obesity and anti-hyperlipidemia		
Dahuang zhechong wan	Liuweidihuang wan	Xuezhikang pian
Danxiang qingzhi keli	Renshen shouwu jiaonang	Xuezhiling pian
Danlou pian	Shanlücha jiangya pian	Xuezhining wan
Dingkun dan	Sangge jiangzhi wan	Xiaokeping pian
Fangfeng tongsheng keli	Songling xuemaikang jiaonang	Yangxinshi pian
Guizhi fuling wan	Shouwu wan	Yindan xinnaotong ruanjiaonang
Hedan pian	Tongxinluo jiaonang	Yixintong pian
Huoxue tongmai pian	Xin^anning pian	Yinxingye jiaonang
Jinshuibao pian	Xinkeshu pian	Zhikang keli
Jiangzhiling pian	Xinxuening pian	Zhimaikang jiaonang
Jiangzhi tongluo ruanjiaonang	Xinyuan jiaonang	Zhengxin jiangzhi pian
Liujunzi wan	Xuefu zhuyu jiaonang	

## Targeting Lipid Metabolism With Bioactive Compounds From TCMs With Anti-Obesity Effects

**FIGURE 1:**
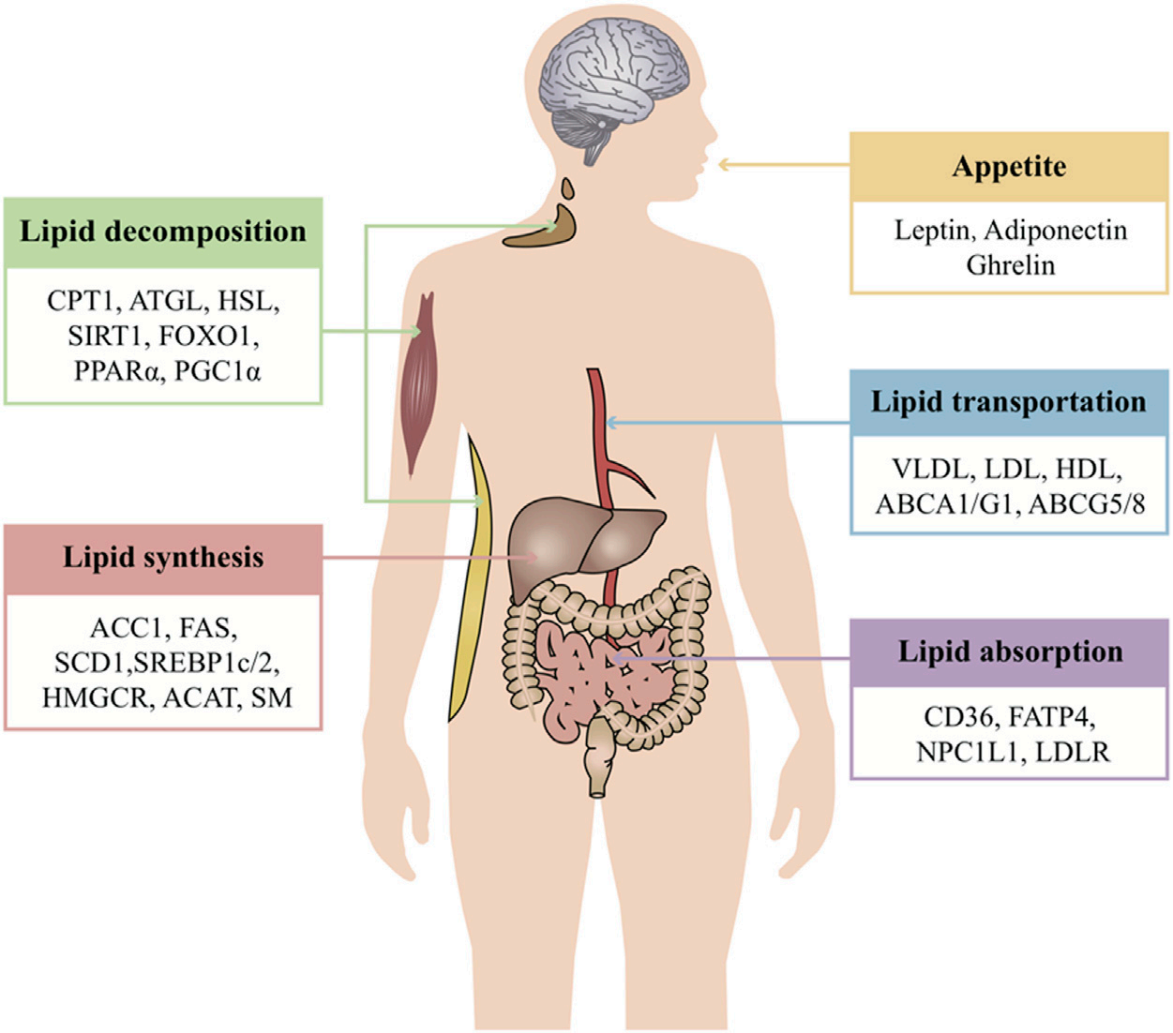
Overview of systematic regulated lipid metabolism of TCMs. TCM treatment of obesity mainly regulates lipid metabolism from five aspects: appetite, lipid absorption, lipid synthesis, lipid decomposition, and lipid transportation. Each link intersects and regulates each other to maintain the stability of the internal environment. One of the advantages of TCM is that it can act on multiple aspects and targets at the same time and systematically regulate life activities. Appetite is regulated by the level of leptin, adiponectin, ghrelin, and so on. These hormones serve as a critical signal to regulate food intake. The small intestine absorbs lipids derived exogenously from the diet. Dietary fat comprises a variety of lipids, while lipids synthesized in the liver are packaged in very low–density lipoproteins and delivered to adipose tissue for storage. CD36, FATP, NPC1L1, and LDLR are the classic regulators of lipid absorption. Apart from lipid absorption, excessive lipid synthesis is another cause of obesity. Some enzymes of lipid synthesis are the key to disease treatment, such as ACC, FAS, SCD, HMGCR, SM, and ACAT. These enzymes may also be regulated by the transcription factors SREBP and LXR. Cytoplasmic lipolysis and lysosomal-mediated autophagy (lipophagy) are two pathways that are known to break down TAGs and CEs in lipid droplets. In this process, ATGL, HSL, and MGL break down TAGs into free fatty acids which then undergo oxidative decomposition in the mitochondria via CPT1 to provide energy. The activity of these enzymes may also be controlled by SIRT1, FOXO1, and PGC1α. Lipoproteins are the major carriers of lipids in circulation. The major forms of lipoproteins are chylomicrons, VLDL, IDL, LDL, and HDL. The lipoproteins are responsible for transportation of FAs and cholesterol. Furthermore, the transportation of cholesterol to the extracellular environment is controlled by ABCA1, ABCG1, and ABCG5/8.

Over many years of observation and research, it has been found that TCMs can regulate all steps of lipid metabolism, targeting multiple pathways, including the modulation of lipid absorption, reduction of lipid synthesis, and increase of lipid decomposition and lipid transportation, as well as the physiological process of appetite regulation (Figure 1). The following is a systematic and comprehensive review of the anti-obesity effect of TCMs by targeting the above-mentioned lipid metabolism.

### Regulation of Appetite

Feelings of hunger and satiety are regulated by complex neural and endocrine interactions among the gut, brain, adipose tissues, and other organs. As early as the 1960s, leptin was identified as a hormone linked to obesity. Leptin is secreted by adipose tissue and regulates appetite through inhibiting orexigenic neurons while stimulating anorexigenic pro-opiomelanocortin neurons (Friedman, 1997; Elias et al., 1999). Another hormone, ghrelin, which is released by the gastrointestinal tract when the stomach is empty, induces hunger by acting on hypothalamic brain cells in the central nervous system (CNS) (Ahima and Antwi, 2008). Moreover, the protein hormone adiponectin is secreted by adipocytes and circulates in the plasma. In contrast to leptin, adiponectin is reduced in obesity and increased in response to fasting. Adiponectin deficiency induces insulin resistance (IR) and hyperlipidemia and is associated with increased susceptibility toward vascular injury and atherosclerosis (Kadowaki et al., 2008).

Discovery of leptin brought hopes for treatment of obesity. Data from both humans and animals have established that the leptin level increases and is positively correlated with fat mass, thereby suppressing appetite. Conversely, weight loss leads to a decrease in the leptin level and produces a consequent increase in food intake (Campfield et al., 1995; Halaas et al., 1995; Pelleymounter et al., 1995; Montague et al., 1997; Licinio et al., 2004; Farooqi et al., 2007). Adiponectin levels decrease in obesity. Adiponectin enhances AMPK activity in the arcuate hypothalamus (ARH) *via* its receptor AdipoR1 to stimulate food intake; this stimulation of appetite by adiponectin is attenuated by the dominant-negative AMPK expression in the ARH (Kubota et al., 2007). Ghrelin levels increase during food deprivation in animals and prior to meals in humans and may serve as a critical signal to induce hunger during fasting (Ahima and Antwi, 2008).

As mentioned, TCMs can affect multiple steps in these hormone signaling pathways. *Salvia miltiorrhiza* Bunge can significantly inhibit the appetite and body weight by increasing the sensitivity to leptin and inhibiting ghrelin activity (Xin-Min et al., 2010; Tung et al., 2017). A high-fat diet (HFD) can increase the serum levels of leptin, insulin, and glucose. *Polygonum multiflorum* Thunb. could reverse these changes (Choi et al., 2018). Administration of *Panax notoginseng* (Burkill) F.H. Chen saponins (PNSs) for 30 days resulted in a significant decrease in serum insulin, leptin, body weight, food intake, and serum triglyceride (TG) levels compared with a diabetic control group (Yang et al., 2010). *Curcuma longa* L. may contribute to decreasing body weight and regulating leptin secretion in animals (Song and Choi, 2016) and humans (Navekar et al., 2017). Baicalin, a flavonoid of the herbal medicine *Scutellaria baicalensis* Georgi, also increased the plasma leptin level vs. the diabetic control (Waisundara et al., 2009). Finally, rats fed with fructose/green tea and fructose/pu-erh tea showed the greatest reduction in serum TG, cholesterol, insulin, and leptin levels (Huang and Lin, 2012). Consistent with obesity induction, adiponectin levels were reduced in HFD-fed mice and adiponectin levels were restored after green tea treatment in the wild type (WT) (Bolin et al., 2020). The green tea polyphenols also have the same curative effect (Tian et al., 2013).

Capsaicin is the molecule that is responsible for the pungency of hot peppers. It functions by stimulating the sympathoadrenal system that mediates the thermogenic and anorexigenic effects of capsaicinoids. Capsaicin treatment in mice fed on an HFD for 10 weeks lowered obesity, fasting glucose, insulin, leptin, and hepatic TGs while increasing adiponectin mRNA/protein in the adipose tissue. Furthermore, capsaicin increased GLP1 and decreased ghrelin secretion, indicating a possible interaction between transient receptor potential cation channel subfamily V member 1 (TrpV1) and GLP1 (Smeets and Westerterp-Plantenga, 2009; Kang et al., 2010). Also, leptin levels in the plasma were significantly lower in resveratrol-treated animals (Jimoh et al., 2018; Yu et al., 2019) and humans (Timmers et al., 2011). Celastrol, a compound of *Tripterygium wilfordii* Hook. f*.*, is a leptin sensitizer (Xu et al., 2021). It can suppress appetite, block the reduction of energy expenditures, and lead up to a 45% weight loss in hyperleptinemic diet–induced obese mice by increasing leptin sensitivity (Liu et al., 2015). Celastrol, an NF-κB inhibitor, reduced IR and lipid abnormalities and led to higher plasma adiponectin levels in the *db/db* mice with celastrol treatment for 2 months (Kim et al., 2013).

### Regulation of Lipid Uptake From the Intestine

**FIGURE 2:**
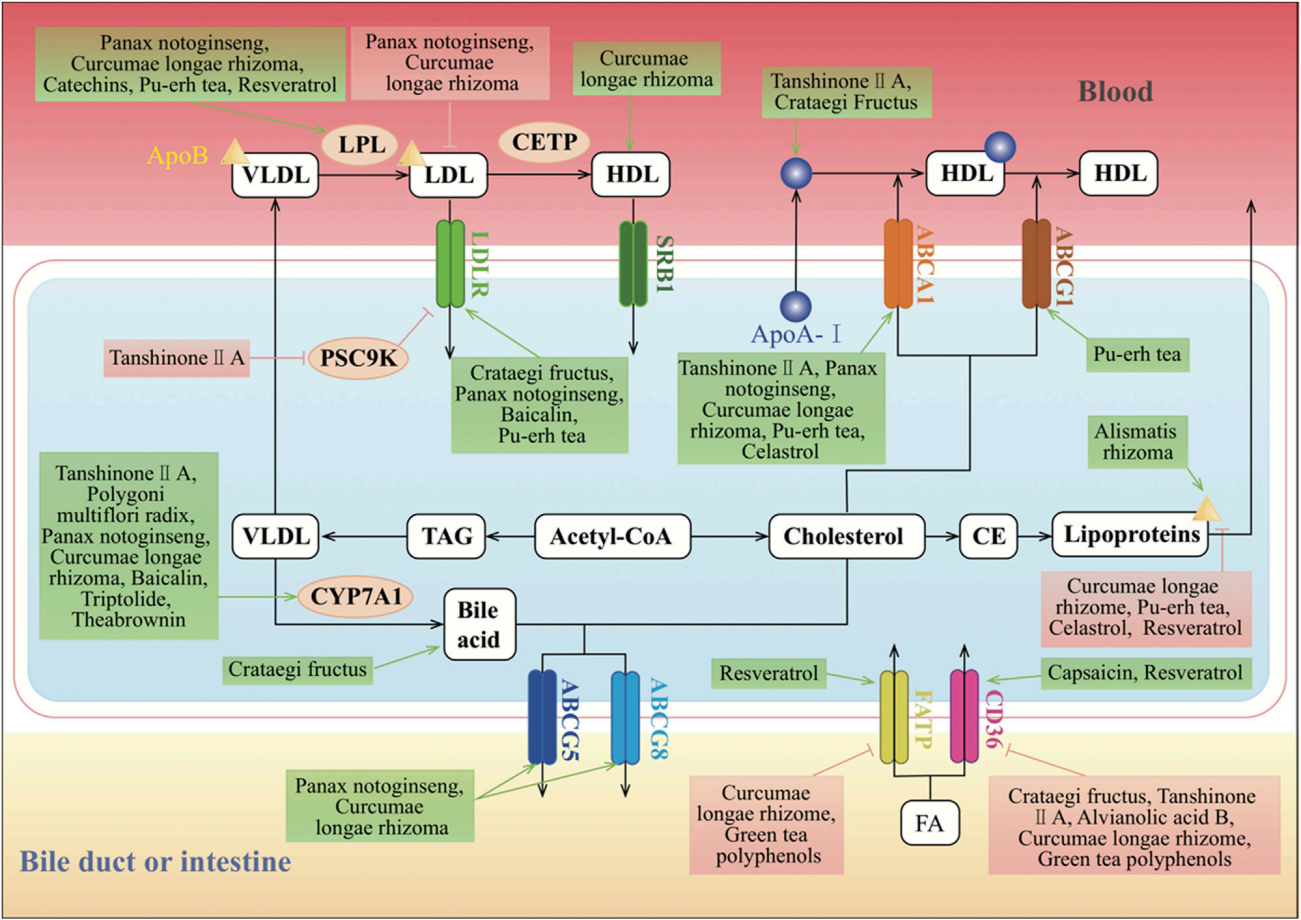
Molecular mechanisms of TCMs in lipid absorption and transportation. CD36, FATP, and LDLR are the classic regulators of lipid absorption. CD36 and FATP mediate the absorption of FA, and LDLR mediates the absorption of LDL. SR-BI can mediate the selective absorption of CE, which plays an important role in HDL metabolism and cholesterol “reversal.” Cholesterol is synthesized from acetyl-CoA. Excess cholesterol in hepatic cells is exported to the blood by ABCA1 or the homodimer of ABCG1, or to the intestinal lumen and bile ducts by the ABCG5 and ABCG8 heterodimers. Cholesterol can also be converted to CE by ACAT for storage in lipid droplets or for secretion as lipoproteins. The major forms of lipoproteins are chylomicrons, VLDL, IDL, LDL, and HDL, and they differ in their size, density, composition, and functions. CYP7A1 converts cholesterol into bile acids in the reverse cholesterol transport pathway. In the endogenous pathway, the liver produces VLDL, which interacts with LPL in the circulation to form IDL, with the release of TG and FAs. IDL is rapidly removed by the liver *via* the interaction of its apolipoprotein E component with LDLR. Furthermore, IDL forms LDL upon removal of TG by hepatic lipase. CETP induces LDL to generate HDL, which is an anti-atherogenic lipoprotein or “good cholesterol,” as it captures the cholesterol from peripheral tissues or other lipoproteins and transports it back to the liver by the third pathway, which is termed reverse cholesterol transport. ↑/⊥ depicts the positive or negative effect of TCMs in the cellular process, respectively.

The small intestine absorbs lipids derived exogenously from the diet including non-polar lipids, predominantly TAGs and CEs, and polar PLs. Dietary lipids such as TAGs, CEs, and PLs along with endogenous lipids from the bile are completely digested by pancreatic enzymes in the intestinal lumen, producing fatty acids (FAs), monoacylglycerols (MAGs), cholesterol, and lysophospholipids (Ko et al., 2020). The uptake of FAs and MAGs can be driven by the concentration gradient or facilitated by other proteins such as cluster of differentiation 36 (CD36) and fatty acid transport protein 4 (FATP4). Cholesterol uptake is mediated by Niemann–Pick C1-like 1 (NPC1L1). The TCMs can regulate lipid(s) uptake from the intestine (Figure 2).

#### Regulation of Uptake of FAs

FAs in the liver originate from the diet, *de novo* lipogenesis (DNL), and recycling of FAs released from adipose tissue during fasting (Mashek, 2013). FAs are taken in the intestinal lumen into enterocytes by two distinct mechanisms. In the first process, FAs diffuse passively through the apical membrane when luminal concentrations are higher than those inside the cell. The other mechanism of FA uptake is saturable and probably protein dependent as this transport occurs when the intracellular FA concentration is higher than that in the lumen.

Tanshinone ⅡA could decrease oxidized low-density lipoprotein (oxLDL)-induced expression of lectin-like oxidized LDL receptor-1 (LOX-1) and CD36 (Wen et al., 2020). And salvianolic acid B inhibited macrophage uptake of modified LDL in a scavenger receptor CD36–dependent manner (Bao et al., 2012). *Curcuma longa* L. suppressed the expression levels of CD36 and FATP, which were increased in HFD groups (Mun et al., 2019). Curcumin, a yellow-colored hydrophobic polyphenol, is the principal curcuminoid of the spice turmeric, the ground rhizome of *Curcuma longa L.*. Curcumin could also downregulate the mRNA level of *Cd36* during adipocyte differentiation of 3T3-L1 cells (Zhao et al., 2011). CD36 mRNA and protein levels were decreased in high-fructose diet–induced rats when treated with green tea polyphenol (Qin et al., 2010). Epigallocatechin gallate (EGCG), a green tea bioactive polyphenol, also dose-dependently reversed HFD-induced effects on intestinal substrate transporters CD36, FATP4, and sodium-dependent glucose transporter 1 (Friedrich et al., 2012). In contrast, capsaicin or capsinoids could upregulate the expression of CD36 (Hong et al., 2015).

#### Regulation of Cholesterol Uptake

Classical cholesterol metabolism studies have confirmed that there are two main sources of cholesterol in the human body: exogenous cholesterol from the diet absorbed in the intestine and reabsorbed in the bile and endogenous cholesterol obtained through *de novo* synthesis from acetyl-CoA by the liver and peripheral tissue.

Exogenous cholesterol enters the enterocytes through NPC1L1 and the associated flotillins present on the apical surface of these cells. Curcumin could lower plasma cholesterol and prevent diet-induced hypercholesterolemia through modulating intestinal NPC1L1 expression *via* transcriptional regulation and the involvement of the sterol regulatory element–binding protein 2 (SREBP2) transcription factor (Kumar et al., 2011). In addition to NPC1L1-mediated cholesterol absorption from the intestinal lumen, another pathway is LDL receptor (LDLR)-mediated uptake of cholesterol containing LDL particles (LDL-c) from the blood. LDL in the blood is captured by LDLR on the cell surface and internalized, and as the endosomal pH decreases, LDLR dissociates from LDL and is recycled back to the surface for additional uptake. LDL is further delivered to lysosomes, and the carried cholesteryl esters (CEs) are hydrolyzed to cholesterol.

Tanshinone ⅡA and *Crataegus pinnatifida* Bunge can regulate the expressions of LDLR in the liver (Hu et al., 2016; Jia et al., 2016b). The *Ldlr* mRNA level was significantly higher in rats by treatment with an *n*-butanol extract (NE3) of *Panax notoginseng* (Burkill) F.H. Chen (Ji and Gong, 2007). The *Scutellaria baicalensis* Georgi extract also activated *Ldlr* genes in the liver. Co-administration of this extract with baicalin and metformin exerted a better effect on obesity-induced IR and lipid metabolism in a rat model system than treatment with metformin alone (Han et al., 2017). Also, proprotein convertase subtilisin/kexin type 9 (PCSK9), a negative regulator of LDLR, is also an SREBP2 target. Tanshinone ⅡA treatment inhibited the expression of PCSK9 and concomitantly increased LDLR activity (Chen et al., 2016).

#### Regulation of Gut Microbiota

The community of microorganisms living in the gastrointestinal tract in animals and humans has been shown to participate in various physiological and pathological processes in the gut and many other bodily processes. The link between the microbes in the human gut and the development of obesity and obesity-related diseases is becoming clearer. Studies have shown differences in the gut microbiota between obese individuals and lean individuals. Obesity and the associated metabolic syndromes are associated with microbiota alterations, including an increase in the ratio of Firmicutes to Bacteroidetes and in the relative abundance of Proteobacteria as well as alterations in specific bacteria such as *Lactobacillus* and *Clostridium* (Ley et al., 2005; Turnbaugh et al., 2006; Fei and Zhao, 2013; Cortés-Martín et al., 2020). There are also a reduced bacterial diversity and altered representations of bacterial genes and metabolic pathways (Turnbaugh et al., 2009; Wu et al., 2021).

Studies on both mice and humans show effects of gut microbiota on lipid metabolism by improving energy extraction from food, which is considered an environmental factor contributing to obesity and its comorbidities (Santacruz et al., 2009; Ridaura et al., 2013). Compared to lean mice, the gut microbiota of the obese mice have an increased capacity to harvest energy from the diet (Turnbaugh et al., 2006). Moreover, when compared with conventional mice, germ-free mice were able to resist obesity on a high-fat, high-carbohydrate Western diet, which could be explained by their intake of fewer calories, increased lipid excretion in the feces, and increased lipid oxidation in the intestine and peripheral tissues (Bäckhed et al., 2007; Rabot et al., 2010). In an observational study using fecal microbiota transplantation, the transplantation of feces from twins discordant for obesity into germ-free mice in a diet-dependent manner demonstrated transmissible, rapid, and modifiable effects of diet-by-microbiota interactions (Ridaura et al., 2013).

Many studies have emerged suggesting that the therapeutic potential of TCMs and their bioactive compounds may be due to the interaction with gut microbiota. Theabrownin, one of the most active and abundant pigments in pu-erh tea, altered the gut microbiota in both mice and humans and increased the levels of ileal conjugated bile acids by predominantly suppressing microbes associated with bile-salt hydrolase (BSH) activity. This in turn inhibited the intestinal farnesoid X receptor (FXR)–fibroblast growth factor 15 (FGF15) signaling pathway that increased hepatic production and fecal excretion of bile acids, thereby reducing hepatic cholesterol and decreasing lipogenesis (Huang et al., 2019). Green tea polyphenols decreased the relative abundance of Bacteroidetes and Fusobacteria and increased the relative abundance of Firmicutes as revealed by 16S rRNA gene sequencing analysis in canines with HFD-induced obesity (Li et al., 2020). The gut microbiota played an important role in the anti-obesity effects of celastrol, in which it promoted energy expenditure at a dose of 500 µg/kg body weight and improved the diversity of the gut microbiota with an increased ratio of Bacteroidetes to Firmicutes (Hu et al., 2020). Capsaicin has an anti-obesity effect through alterations in gut microbiota populations and short-chain FA concentrations, which were beneficial in prevention and treatment of obesity (Song et al., 2017; Rosca et al., 2020; Wang Y. et al., 2020). Resveratrol-induced gut microbiota modulated lipid metabolism, stimulated the development of beige adipocytes in white adipose tissue, reduced inflammation, and improved intestinal barrier function in HFD-fed mice. Therefore, the anti-obesity benefits of resveratrol might be through the “gut microbiota–adipose tissue” axis (Wang P, et al., 2020; Zhou et al., 2019).

### Regulation of Lipid Transportation

Lipoproteins are the major carriers of lipids in circulation and participate in three major pathways that are responsible for the generation and transport of lipids within the body. The two major forms of circulating lipids in the body, TG and cholesterol, are packaged with apolipoproteins and PLs to form lipoproteins. The major forms of lipoproteins are chylomicrons, very low–density lipoprotein (VLDL), intermediate-density lipoprotein (IDL), low-density lipoprotein (LDL), and high-density lipoprotein (HDL), which differ in their size, density, composition, and functions.

In the exogenous pathway, dietary lipids, which mainly consist of TGs and some PLs, free FAs, and cholesterol, are packaged into chylomicrons by intestinal mucosal cells. These chylomicrons enter the lymphatic system and then the circulation, where TGs are released as free FAs by lipoprotein lipase (LPL) activity on the capillary endothelium. These free FAs are taken up by the muscle, adipose, and other peripheral tissues, whereas the remnants of chylomicrons are cleared by the liver. In the endogenous pathway, the liver produces VLDL, which interacts with LPL in the circulation to form IDL, with the release of TGs and free FAs. IDL is rapidly removed by the liver *via* the interaction of its apolipoprotein E component with LDLR. Furthermore, IDL forms LDL upon removal of TGs by hepatic lipase (HL). LDL, which is very high in cholesterol content, is in turn removed from the circulation by binding to LDLR in the liver and in extrahepatic tissues. HDL is an anti-atherogenic lipoprotein or “good cholesterol,” as it captures the cholesterol from peripheral tissues or other lipoproteins and transports it back to the liver by the third pathway, which is termed reverse cholesterol transport (Luo et al., 2020) (Figure 2).

#### Regulation of Lipoprotein Uptake

The liver has a role in the regulation of systemic lipid metabolism as it assembles and secretes TAG-rich VLDL particles into the systemic circulation for distribution of FAs to the peripheral tissues. Surface LDLR captures the circulating LDL *via* the extracellular ligand–binding domain. Many results suggest that green tea polyphenols inhibit the ubiquitin/proteasome-mediated upregulation of LDLR. This identified molecular mechanism might be related to the previously reported cholesterol-lowering and heart disease–preventative effects of green tea polyphenols (Kuhn et al., 2004). Trans-resveratrol exhibited the anti-atherogenic effect, at least, in part, by increased hepatic LDLR expression *via* proteolytic activation of SREBPs and subsequent LDL uptake (Yashiro et al., 2012).

By contrast, HDLs are generated by the intestine and the liver through the secretion of lipid-free apolipoprotein A-I (ApoA-I). ApoA-I then recruits cholesterol from these organs through the actions of ATP-binding cassette transporter A1 (ABCA1), forming nascent HDLs. In the peripheral tissues, nascent HDLs promote the efflux of cholesterol from tissues, including from macrophages, through the actions of ABCA1. Mature HDLs also promote this efflux, but through the actions of ATP-binding cassette transporter G (ABCA) 1. Tanshinone ⅡA reduced the lipid deposition in the liver. Moreover, it did not affect the serum lipid levels but reduced the levels of HDL middle subfractions and increased the levels of HDL large subfractions (Jia et al., 2016b). Treatment of THP-1 macrophages with baicalin significantly accelerated HDL-mediated but not ApoA-I–mediated cholesterol efflux. However, baicalin treatment increased the expression of scavenger receptor class B type I (SR-BI) in a dose- and time-dependent manner. Furthermore, baicalin increased the expression of peroxisome proliferator–activated receptor (PPAR) *γ*, a key regulator of reverse cholesterol transport, and liver X receptor (LXR) *α* (Yu et al., 2016). Administration of *Curcuma longa* L. significantly decreased the serum LDL and ApoB but increased the serum HDL and ApoA of healthy subjects (Ramirez-Bosca et al., 2000). Resveratrol treatment after 6 months decreased LDL-c, ApoB, oxLDL, and oxLDL/ApoB on statin-treated patients in primary cardiovascular disease prevention (Tome-Carneiro et al., 2012). The LPL was increased significantly in muscular tissues and decreased in adipose tissues by treatment with *Crataegus pinnatifida* Bunge flavonoids in mice (Fan et al., 2006). Finally, PNSs could also upregulate the mRNA expression of *Lpl* (Wang et al., 2016).

#### Regulation of Cholesterol Efflux

Cholesterol export from cells is mediated by ATP-binding cassette transporters. ABCA1 is expressed on the plasma membrane of most cells, including the basolateral surface of enterocytes. ABCG1 is most abundantly expressed on the surface of macrophages, whereas ABCG5 and ABCG8 are expressed at the apical surface of enterocytes and hepatocytes, forming a heterodimer. Excess cholesterol is esterified by acyl coenzyme A–cholesterol acyltransferases (ACATs). ABCA1 mediates cholesterol transport to ApoA-I in the blood, and this generates a nascent HDL that serves as an acceptor for ABCG1-mediated cholesterol efflux, leading to the production of HDL.

Tanshinone IIA treatment suppressed the expression of *miR-33a*, an ABCA1 negative regulator, whereas it upregulated the expression levels of ABCA1, SREBP2, PCSK9, cholesterol 7α-hydroxylase (CYP7A1), CD36, and LDLR in hyperlipidemic rats (Jia et al., 2016a; Jia et al., 2016b). The expression of PPARα and ApoA-Ⅰ was significantly downregulated in the hyperlipidemia group with tanshinone IIA treatment (Yi-Xin et al., 2017). A high dose of *Crataegus pinnatifida* Bunge increased the expressions of ApoA-I gene and HDL-c in HFD-fed mice (Shih et al., 2013). In addition, curcumin increased cholesterol efflux by activating and upregulating the expression of LXR and ABCA1 in subcutaneous adipocytes isolated from rabbits (Dong et al., 2011). The *Curcuma longa* L. oil treatment significantly increased the hepatic expression of PPARα, LXRα, CYP7A1, ABCA1, ABCG5, ABCG8, and LPL accompanied by a reduced SREBP2 and 3-hydroxy-3-methylglutaryl coenzyme A reductase (HMGCR) expression. *Curcuma longa* L. oil treatment also suppressed NPC1L1 expression in the jejunum compared with high-cholesterol diets (Singh et al., 2013). The expression of the reverse cholesterol transporters ABCA1 and ABCG1 was highly expressed in the livers of mice on pu-erh tea intervention (Huang et al., 2019). Furthermore, apolipoprotein B100 (ApoB100) is a constitutive protein of LDL-c, and it was significantly downregulated by pu-erh tea extract (PTE) treatment (Hu et al., 2017).

Cholesterol and sitosterol can be exported by the ABCG5–ABCG8 heterodimers to the intestinal lumen and bile, where cholesterol is extracted by bile salts. Re-synthesis of cholesterol induces pathways for cholesterol export and storage and acts to suppress further cholesterol biosynthesis. When treated with NE3, the concentrations of serum TC, TG, and LDL-c in rats showed a significant dose-dependent decrease. Expression level analysis indicated that both LXR targeting genes including ABCA1, ABCG5, and ABCG8 and FXR targeting genes including ApoC2 and a short heterodimer partner were significantly induced by NE3 (Ji and Gong, 2007). In addition, CE combines with ApoB to form lipoproteins, which are transported outside the cell by exocytosis. *Curcuma longa* L., resveratrol, and PTE could significantly decrease the level of ApoB (Ramirez-Bosca et al., 2000; Tome-Carneiro et al., 2012; Hu et al., 2017). Celastrol was able to effectively suppress weight and attenuate high-fat–mediated oxidative injury by improving ABCA1 expression, reducing the levels of TC, TG, LDL-c, and ApoB in the plasma, and increasing antioxidant enzyme activities and inhibiting nicotinamide adenine dinucleotide phosphate (NADPH) oxidase activity (Wang C. et al., 2014).

Furthermore, CYP7A1 regulates the balance between cholesterol supply and metabolism by catalyzing the rate-limiting step of bile acid biosynthesis. *Scutellaria baicalensis* Georgi activated the *Cyp7a1* gene in the liver (Han et al., 2017). *Crataegus pinnatifida* Bunge could counteract the downregulation of CYP7A1 and LDLR with the stimulation effect of HFD (Hu et al., 2016). And NE3 significantly decreased the expression level of the *Cyp7a1* gene (Ji and Gong, 2007). In contrast, the expression of *Cyp7a1* was markedly increased in the triptolide-treated group (Yang et al., 2017).

### Regulation of Lipid Synthesis

**FIGURE 3:**
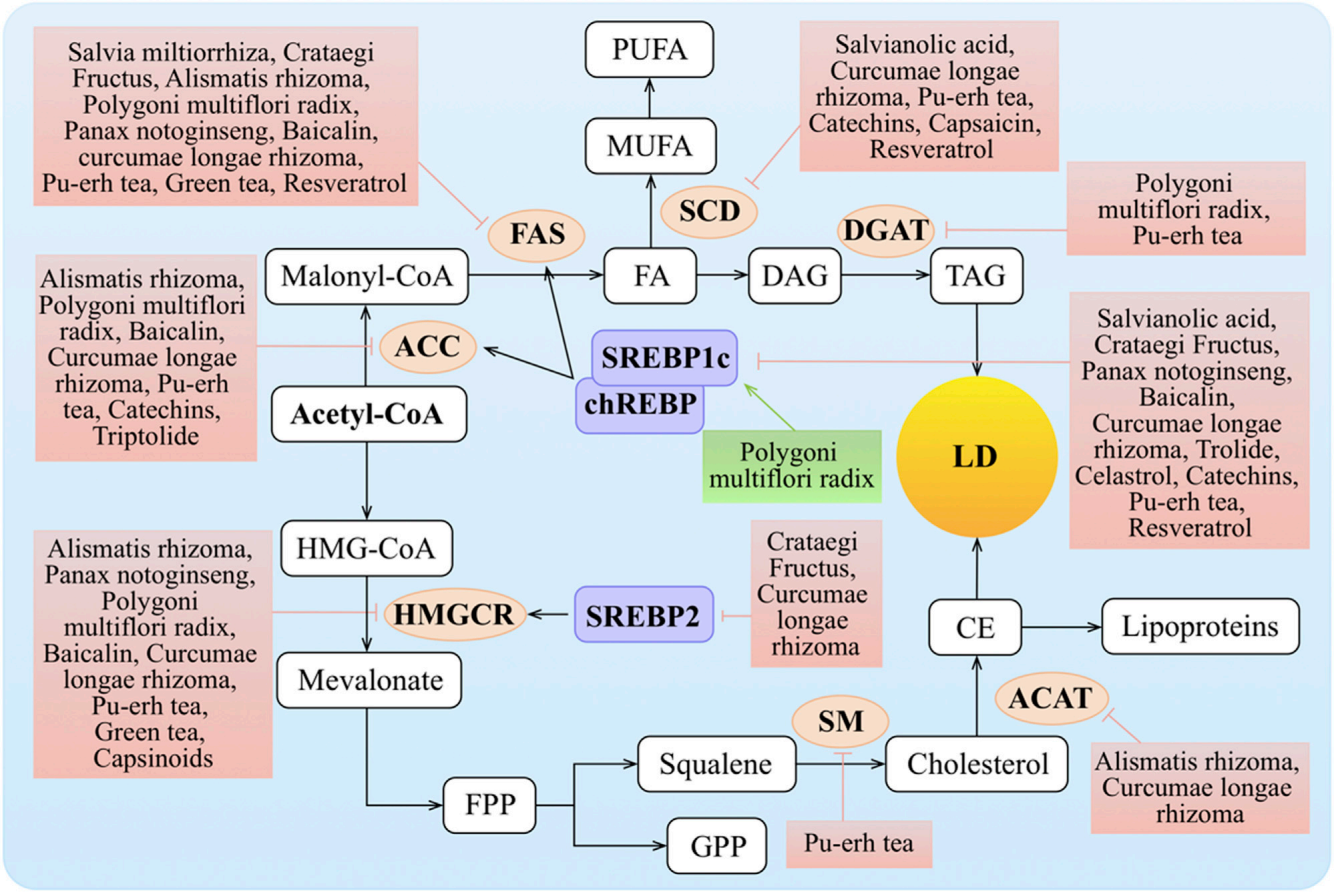
Molecular mechanisms of TCMs in lipid synthesis. FA entering the hepatocyte is rapidly “activated” by acyl-CoA, and this is also termed the *de novo* lipogenesis (DNL) of FA. DNL is mainly regulated *via* the rate-limiting enzymes such as ACC and FAS, while the expression and activation of ACC and FAS are regulated by SREBP1c and chREBP. Transcription of the genes encoding SREBP1c and chREBP is stimulated by insulin *via* LXR and inhibited by FAs. The synthesis of MUFA regulates by SCD. FAs are typically esterified to TG and subsequently packaged into VLDL for export or stored as intracellular LDs. HMGCR and SM as the rate-limiting enzymes are key regulators of the synthesis process of cholesterol. Cholesterol can be converted to CE by ACAT for storage in LDs or for secretion as lipoproteins. Meanwhile, SREBP2 can increase the level of HMGCR. ↑/⊥ depicts the positive or negative effect of TCMs in the cellular process, respectively.

Excessive lipid synthesis is another cause of obesity and hyperlipidemia. Lipids synthesized in the liver are packaged in very low–density lipoproteins (VLDLs) and delivered to adipose tissue for storage. Clearly, TCMs play important roles in lipid synthesis, including FA and TAG synthesis as well as cholesterol biosynthesis (Figure 3).

#### Regulation of Fatty Acid Synthesis

The DNL of FAs from acetyl-CoA to fatty acyl-CoA is mainly regulated by acetyl-CoA carboxylase (ACC) and fatty acid synthase (FAS) as the rate-limiting enzymes. Stearoyl-CoA desaturase (SCD) is a central enzymatic node in the conversion of saturated fatty acids (SFAs) into mono-unsaturated fatty acids (MUFAs) (AM et al., 2017). MUFAs represent the precursors of several lipids essential for plasma membranes, such as TGs, CEs, diacylglycerols, and wax esters. Transcriptional regulation of *Acc* and *Fas* is primarily through SREBP1c and carbohydrate-responsive element–binding protein (chREBP). SCD expression is regulated by diverse hormonal and nutritional factors and is positively regulated by *Srebp1c*, *chrebp*, and *Lxr* (Wang Y., et al., 2015). Both transcription factors are activated by *Lxr*.

Salvianolic acids, the major water-soluble ingredients of *Salvia miltiorrhiza* Bunge, reduced ovariectomy-induced body weight gain, attenuated the expressions of hepatic lipogenic genes, such as *Srebp1*, *Fas*, and *Scd*, and decreased the TG and TC *via* blocking signal transducer and activator of transcription (STAT)-3/SREBP1 signaling (Chen et al., 2018). The hepatic *Fas* and *Srebp1c* mRNA levels were reduced in mice fed on the *Crataegus pinnatifida* Bunge diet compared to the standard diet (Zhang et al., 2014). The levels of FAS and ACC in the plasma were generally reduced after administration of *Polygonum multiflorum* Thunb. (Xian et al., 2017). *Alisma plantago-aquatica* L. inhibited adipocyte differentiation by downregulating the expression of PPAR*γ*, CCAAT/enhancer-binding protein *β* (C/EBPβ), and FAS (Park et al., 2014) and blocked hepatic lipid production by regulating hepatic lipogenic genes including *Fas*, *Acc*, and glycerol-3-phosphate acyltransferase (GPAT) (Choi et al., 2019). Alisol B 23-acetate, a natural triterpenoid isolated from *Alisma plantago-aquatica* L., decreased hepatic lipogenesis through decreasing hepatic levels of SREBP1c, FAS, ACC, and SCD (Meng et al., 2017). *Panax notoginseng* (Burkill) F.H. Chen could change the fat and inflammation of liver tissue through decreasing expression levels of SREBP1c, ACC, and FAS (Ji and Gong, 2007; Yan-Xia et al., 2011; Zhang et al., 2020). The expression levels of SREBP1c, ACC, and FAS were downregulated in the *Curcuma longa* L. groups compared to the HFD groups (Ejaz et al., 2009; Ahn et al., 2010; Zhao et al., 2011; Mun et al., 2019). Baicalin treatment significantly attenuated methionine and choline–deficient diet (MCD)–induced hepatic lipid accumulation partly through regulating the expression of SREBP1c, FAS, and ACC (Xi et al., 2015; Zhang J. et al., 2018). Capsaicin inhibited the early adipogenic differentiation, lipogenesis, and maturation of adipocytes with concomitant repression of PPARγ and SCD (Ibrahim et al., 2015). The PTE intake tended to decrease *Srebp1c*, *Acc*, and *Fas* mRNA expressions in the liver of the mice (Shimamura et al., 2013), and PTE also downregulated *Scd* and *Srebp* in *Caenorhabditis elegans* to suppress fat accumulation (Ding et al., 2015; Hu et al., 2017). On treatment with green tea, the expression of lipogenesis-related genes *Acc*, *Fas*, and *Scd* was downregulated in the liver (Kim et al., 2009; Friedrich et al., 2012). The transcriptional activities of Srebp1c and forkhead box protein O1 (FOXO1) were significantly decreased by EGCG (Kim et al., 2010). Resveratrol exerted anti-obesity effects *via* mechanisms involving downregulation of ACC, FAS (Alberdi et al., 2011), and SCD (Zhang et al., 2012) and upregulation of the key adipogenic gene *Srebp1c* (Kim et al., 2011; Khaleel et al., 2018) in an HFD model. Celastrol decreased hepatic SREBP1 expression (Zhang et al., 2017). However, triptolide treatment increased the expression of LXR and its target gene, *Srebp1*, in both male and female rats and increased the expression of ACC only in the female rats (Jiang et al., 2016).

#### Regulation of TAG Synthesis

TAG production can come from exogenous FAs in the circulation or intracellular FAs generated by glycolysis and lipogenesis from glucose supplied by excess dietary intake. TAG synthesis is catalyzed by diacylglycerol acyltransferase (DGAT) in the last biosynthesis step. *Polygonum multiflorum* Thunb. supplementation significantly downregulated the expression of *Ppar*γ and *Dgat2* genes in obese mice (Choi et al., 2018).

#### Regulation of Cholesterol Biosynthesis

An increased level of LDL-c and/or TC is a pronounced phenotype of dyslipidemia. Ultimately, it is due to elevated cholesterol. Cholesterol plays an important role in human physiological functions. Almost all cells can synthesize cholesterol, and in this process, three crucial players of the cholesterol biosynthetic pathway are required, SREBP2, which functions as a master transcriptional regulator of cholesterol biosynthesis, and two rate-limiting enzymes of the biosynthetic pathway: HMGCR and squalene monooxygenase (SM).

##### Regulation of HMGCR and ACAT

As the rate-limiting enzyme for cholesterol biosynthesis, HMGCR is highly regulated at the transcriptional, translational, and post-translational levels (Goldstein and Brown, 1990). The formation of CE is another important means of preventing the accumulation of free cholesterol in cells, as this ACAT-mediated pathway directs the storage or secretion of cholesterol.

*Crataegus pinnatifida* Bunge could suppress the stimulation effect of HFD on the transcription of *Hmgcr*, and the transcriptional activity of the *Hmgcr* promoter was inhibited by *Crataegus pinnatifida* Bunge in a dose-dependent manner (Hu et al., 2016). HMGCR was generally reduced after administration of *Polygonum multiflorum* Thunb. (Xian et al., 2017). *Alisma plantago-aquatica* L. showed comparatively high inhibition against ACAT and HMGCR activities in rat livers (Choi et al., 2019). *Panax notoginseng* (Burkill) F.H. Chen also reduced the levels of hepatic HMGCR in HFD rats (Xia et al., 2011).

In addition, relative to the HFD control group, hamsters fed a curcumin-supplemented HFD had lower hepatic cholesterol and TG levels and HMGCR and ACAT activities, along with an increased FA β-oxidation activity (Jang et al., 2008). *Scutellaria baicalensis* Georgi, pu-erh tea, or green tea intervention repressed the expression of HMGCR in the liver (Yamashita et al., 2016; Han et al., 2017). However, capsinoids significantly increased HMGCR in the liver (Hong et al., 2015). Treatment with theabrownin, one of the most active and abundant pigments in pu-erh tea, increased two bile acid synthetic genes, *Cyp7a1* and *Cyp7b1*, in HFD-treated mice (Zeng et al., 2015; Huang et al., 2019).

### Regulation of Lipid Decomposition

**FIGURE 4:**
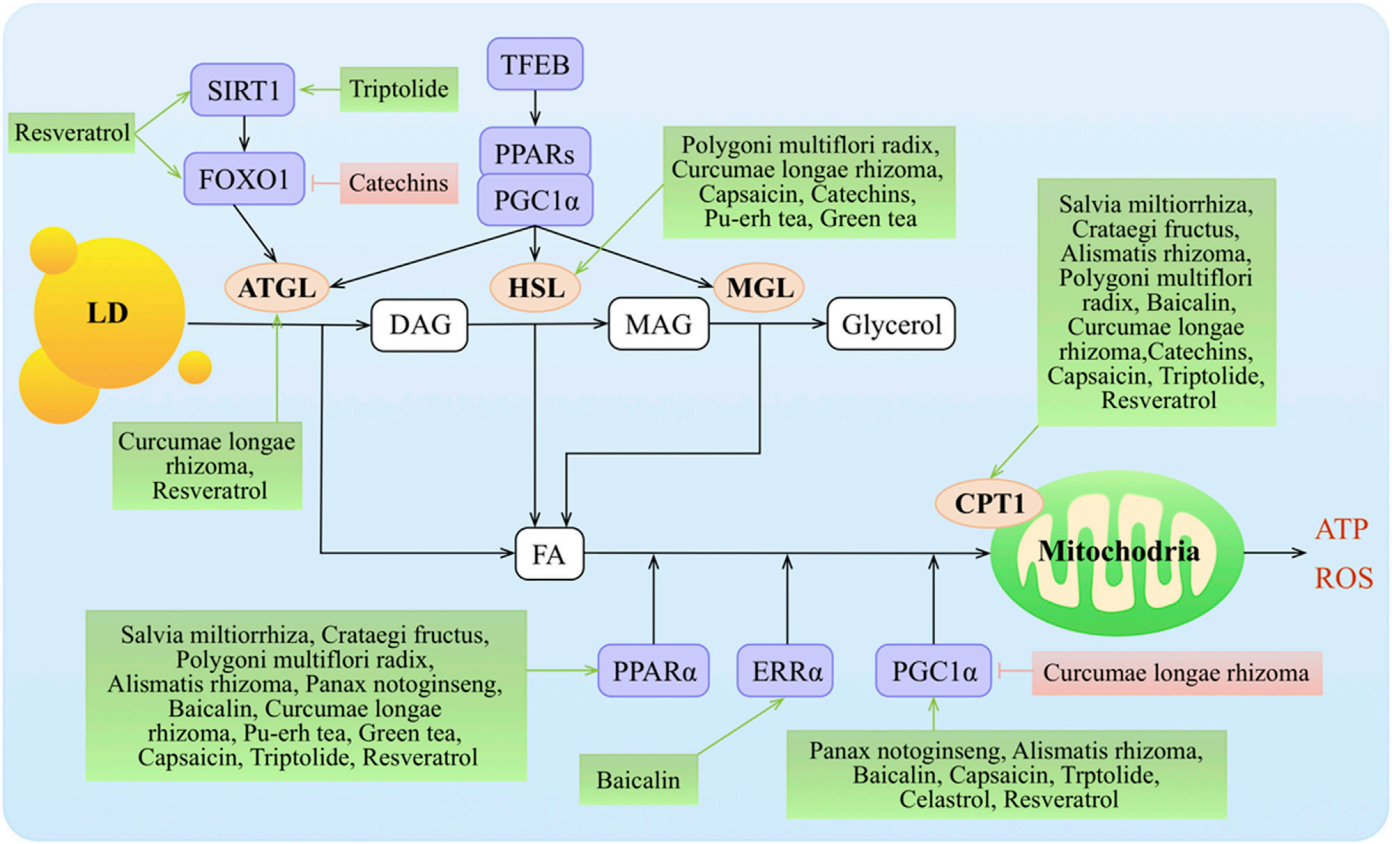
Molecular mechanisms of TCMs in lipid decomposition. TCMs stimulate lipolysis from fat stores in the liver, white adipose tissue, and dietary fat sources (high-fat diets) to generate FAs that enter the hepatic cells *via* protein transporters. TG stored as lipid droplets can be hydrolyzed back to FAs *via* classic lipases (ATGL, HSL, and MGL) and lipophagy (by regulating TFEB, SIRT1, and FOXO1), undergo mitochondrial β-oxidation by the activity of various co-activators or nuclear receptors (such as PPARα, ERRα, and PGC1α), and target the transcription of gene Cpt1. Malonyl-CoA, an intermediate in DNL, inhibits CPT1 action and downregulates FA oxidation. ↑/⊥ depicts the positive or negative effect of TCMs in the cellular process, respectively.

Cytoplasmic lipid droplets (LDs) are multiprotein-coated structures that serve as dynamic TAG storage pools and are involved in several aspects of lipid metabolism. The LDs are mainly rich in TAGs and CEs. There are two pathways that are known to break down TAGs in LDs: cytoplasmic lipolysis and lysosomal-mediated autophagy (lipophagy). Adipose triglyceride hydrolase (ATGL) plays a key role in the lipolysis pathway, breaking down TAGs into diglycerides (DAGs) and FAs. FAs further undergo oxidative decomposition in the mitochondria *via* carnitine palmitoyl transferase 1 (CPT1) and provide energy (Figure 4).

#### Regulation of LD Decomposition

ATGL initiates TAG hydrolysis to form diacylglycerol and FAs. Hormone-sensitive lipase (HSL) (Lafontan and Langin, 2009; Rodriguez et al., 2010) and monoacylglycerol lipase (MGL) complete the process by consecutively hydrolyzing diacylglycerols into MAGs and FAs and then hydrolyzing MAGs into glycerol and FAs (Zechner et al., 2017). Transcription of *Atgl* is controlled by sirtuin 1 (SIRT1)–mediated deacetylation of FOXO1 and by PPARγ co-activator 1α (PGC1α) (Chakrabarti et al., 2011; Chen et al., 2021). The PPAR–PGC1α axis also regulates the transcriptional expression of *Hsl* (Albert et al., 2014; Farhan et al., 2014) and *Mgl* (Rakhshandehroo et al., 2007).

Curcumin treatment upregulated the expression of ATGL and resulted in acceleration of lipolysis (Valentine et al., 2019). Pu-erh tea administration significantly lowered plasma TC and TG concentrations and the LDL-c level but did not affect HDL-c levels. Moreover, pu-erh tea significantly increased LPL, HL, and HSL activities in epididymal fat tissue in rats with HFD-induced obesity (Cao et al., 2011). Resveratrol acted mainly on ATGL to regulate lipolytic activity in humans and murine adipocytes (Lasa et al., 2012) and increased *Sirt1*, *Foxo1*, and adiponectin mRNA expressions (Costa Cdos et al., 2011; Timmers et al., 2011; Lasa et al., 2012).

##### Regulation of *β*-Oxidation of FAs

*β*-Oxidation in the mitochondria is the predominant oxidative pathway for energy production in the liver. *β*-Oxidation consists of a cycling process involving dehydrogenation, hydration, dehydrogenation, and acylation that produces acetyl-CoA. In this process, the most important enzyme is CPT1 (Houten et al., 2016). The primary regulators of *β*-oxidation are the transcription factors *Ppar*α and *Pgc1*α, whose action is upregulated by FAs and glucagon and suppressed *via* insulin (Pawlak et al., 2015).

The expression of PPARα was significantly downregulated in a hyperlipidemia group with tanshinone IIA treatment in rats (Yi-Xin et al., 2017). CPT1, PPARα, and its downstream targets were activated with hawthorn leaf flavonoids in an HFD model (Kuo et al., 2009; Li et al., 2015; Dong et al., 2017). Alisol B 23-acetate administration increased lipid metabolism *via* inducing PPARα, CPT1α, and LPL (Meng et al., 2017). *Alisma plantago-aquatica* L. suppressed the mRNA levels of hepatic *Pgc1*α, estrogen-related receptor (ERR)γ, and PGC1α-dependent enzyme (G6Pase) that are involved in gluconeogenesis in liver tissue (Jeong Hyang Sook %J Korea Journal of Herbology, 2013). *Polygonum multiflorum* Thunb. supplementation significantly upregulated the *Pparα*, *Cpt1*, *Cpt2*, *Ucp1*, and *Hsl* mRNA levels compared with the HFD group (Choi et al., 2018). PNSs regulated lipid metabolism by upregulating the expression of transcriptional factors, such as *Pparα*, *Pparγ*, and *Pgc1α* (Wang et al., 2016). PPARα and CPT1 expressions were upregulated in the *Curcuma longa* L.–treated groups (Mun et al., 2019). Finally, *Curcuma longa* L. treatment in high-fructose diet (HFrD)-fed rats repressed hepatic expression of PGC as compared to the rats fed an HFrD alone, suggesting a protective effect of *Curcuma longa* L. by modulating the expression of lipogenic genes in the liver (Singh et al., 2015). Treatment with baicalin ameliorated diet-induced obesity through directly activating hepatic CPT1 as well as increasing the expression of PPARα (Zhang J. et al., 2018), ERRα, and PGC1α (Takizawa et al., 2015; Dai et al., 2018).

Treatment with capsinoids significantly increased the expression of CPT1, adiponectin, and *Pparα* and *Pgc1α* mRNA in the liver (Kang et al., 2010; Lee et al., 2013; Hong et al., 2015; Panchal et al., 2018). The expression of PPARα was also increased in the HFD/pu-erh tea groups (Sun et al., 2019). Green tea increased the expression of CPT1 and PPARα and decreased the expression of LXR (Chen et al., 2009; Kim et al., 2009; Axling et al., 2012) while increasing HSL in mesenteric adipose tissue concomitantly with the HFD (Cunha et al., 2013). Resveratrol increased CPT1 activities (Gomez-Zorita et al., 2012), had a higher agonistic activity of PPARα (Takizawa et al., 2015), and significantly increased SIRT1 and PGC1α levels and citrate synthase activity and improved muscle mitochondrial respiration (Timmers et al., 2011). Finally, celastrol augmented PGC1α expression in adipocytes and skeletal muscles (Fang et al., 2019).

## Conclusions and Perspectives

Human obesity is quickly becoming widespread, and treatment of it and its comorbidities is an important clinical challenge. Targeting lipid metabolism as a potential treatment has attracted a great deal of attention as a primary therapeutic target. TCMs have been widely used as anti-obesity treatments for a very long time. In this review, how TCMs modulate major features of lipid metabolism was systematically summarized. Collation and integration of these data has produced a comprehensive register for the mechanisms of TCMs’ action in anti-obesity (Table 2).

**TABLE 2:** A summary of studies demonstrating the effects of TCM on lipid metabolism in animal models and humans.

TCM	Appetite	Lipid absorption	Lipid transportation	Lipid synthesis	Lipid decomposition
*Crataegus pinnatifida* Bunge		CD36↓ LDLR↑	ApoA-Ⅰ↑	FAS↓ SREBP-1c↓ SREBP2↓	CPT1↑ PPARα↑
*Salvia miltiorrhiza* Bunge/tanshinone Ⅱ A/salvianolic acid B	Leptin sensitivity↑ Ghrelin↓	CD36↓ LDLR↓ PCSK9↓	ABCA1↑ CYP7A1↑ ApoA-Ⅰ↑	FAS↓ SCD↓ SREBP-1c↓	CPT1↑ PPARα↑
*Polygonum multiflorum* Thunb.	Leptin↓		CYP7A1↑	ACC1↓ FAS↓ DGAT↓ SREBP-1c↑ HMGCR↓;	HSL↑ CPT1↑ PPARα↑
*Alisma plantago-aquatica* L.	Leptin↓		ApoB↑	ACC1↓ FAS↓ DGAT↓ HMGCR↓ HMGCR↓ ACAT↓	CPT1↑ PPARα↑ PGC1α↑
*Panax notoginseng* (Burkill) F.H. Chen/*Panax notoginseng* saponins/*n*-butanol extract of *Panax notoginseng*	Leptin↓	LDLR↑	ABCA1↑ ABCG5/8↑ CYP7A1↑ LPL↑ LDL↓	FAS↓ SREBP-1c↓ HMGCR↓	PPARα↑ PGC1α↑
*Curcuma longa* L./curcumin	Leptin↓	CD36↓ FATP↓ NPC1L1↓	ABCA1↑ ABCG5/8↑ CYP7A1↑ LPL↑ LDL↓ HDL↑ ApoB↓	ACC1↓ FAS↓; SCD↓ SREBP-1c↓ SREBP2↓ ACAT↓ HMGCR↓	CPT1↑ ATGL↑ HSL↑ PGC1α↓ PPARα↑
*Scutellaria baicalensis* Georgi/baicalin		LDLR↑;	CYP7A1↑	ACC1↓ FAS↓ SREBP-1c↓ HMGCR↓	CPT1↑ PPARα↑ ERRα↑ PGC1α↑
Pu-erh tea/theabrownin	Leptin↓		ABCA1↑ ABCG11↑ LPL↑ ApoB↓	ACC1↓ FAS↓ SCD↓ DGAT↓ SREBP-1c↓ HMGCR↓ SM↓	HSL↑ PPARα↑
Green tea/green tea polyphenols/EGCG	Leptin↓ Adiponectin↑	CD36↓ FATP↓	LPL↑	FAS↓ ACC1↓ SCD↓ HMGCR↓	HSL↑ FOXO1↓ CPT1↑ PPARα↑
*Tripterygium wilfordii* Hook. f./celastrol/triptolide	Leptin sensitivity↑ Adiponectin↑		ABCA1↑ ApoB↓ CYP7A1↑	ACC1↓ SREBP-1c↓	CPT1↑; PPARα↑; PGC1α↑; SIRT1↑;
Capsaicin	Leptin↓ Adiponectin↑ ghrelin↓	CD36↑		SCD↓ HMGCR↓	HSL↑ CPT1↑ PPARα↑ PGC1α↑
Resveratrol	Leptin↓	CD36↑ FATP↑	LPL↑ ApoB↓;	FAS↓ SCD↓ SREBP-1c↓	ATGL↑ SIRT1↑ FOXO1↑ CPT1↑ PPARα↑ PGC1α↑

↑: increase; ↓: decrease.

While TCMs clearly play roles in anti-obesity, the side effects of TCMs should not be overlooked. Hepatotoxicity is the main side effect of TCMs (Tarantino et al., 2009; Frenzel and Teschke, 2016). In many cases, the side effects are caused by incorrect processing, usage, dosage, or compatibility. For example, the use of the processed *Polygonum multiflorum* Thunb. caused less damage to the liver than the unprocessed *Polygonum multiflorum* Thunb. (Tu et al., 2015), while EGCG caused dose-dependent hepatotoxicity in mice under dietary restriction, but not in mice fed a normal diet (Shi et al., 2020). In addition, *Tripterygium wilfordii* Hook. f*.* is normally hepatotoxic, but the classical compatibility of *Tripterygium wilfordii* Hook. f. and *Lysimachia christinae* Hance can detoxify the poison of *Tripterygium wilfordii* Hook. f*.* (Wang et al., 2018; Wang J., et al., 2015). Triptolide and celastrol are two major components of *Tripterygium wilfordii* Hook. f.. Interestingly, triptolide is hepatotoxic, while celastrol showed protection from liver injury (Hasnat et al., 2019; Xu et al., 2021). These cases suggest that correct processing, usage, dosage, and compatibility under the application guidance based on long experience can greatly reduce side effects. Therefore, for the use of TCMs in anti-obesity, following the doctor’s advice and guidelines of the TCMs is essential to ensure the efficacy of the TCMs and also avoid side effects as much as possible.

Moreover, the capacity of TCMs to inhibit obesity is attracting increasing attention. TCMs are being advocated as another major breakthrough for therapeutic intervention for obesity-related diseases. However, only a fraction of the medically active substances available in TCMs have been identified, and the unidentified natural products have great potential. Modern technologies enable the detailed analysis of TCM extracts to identify active substances. These phytochemicals, in the form of the TCMs themselves, extracts, or purified components, can be combined with existing treatments to reduce the prevalence of obesity and its complications. Taking advantage of TCM effects on therapeutic interventions for the treatment of obesity-related diseases may be another breakthrough for integrated medicine.

## Glossary

ABCA: ATP-binding cassette transporter A
ABCG: ATP-binding cassette transporter G
ACAT: acyl coenzyme A–cholesterol acyltransferase
ACC: acetyl-CoA carboxylase
ApoA: apolipoprotein A
ARH: arcuate hypothalamus
AS: atherosclerosis
ATGL: adipose triglyceride hydrolase
BMI: body mass index
BSH: bile-salt hydrolase
C/EBPβ: CCAAT/enhancer-binding protein β
CD36: cluster of differentiation 36
chREBP: carbohydrate-responsive element–binding protein
ChP: Pharmacopoeia of the People's Republic of China
CNS: central nervous system
CPT1: carnitine palmitoyl transferase 1
CEs: cholesterol esters
CVD: cardiovascular disease
CYP7A1: cholesterol 7α-hydroxylase
DGAT: diacylglycerol acyltransferase
DNL: de novo lipogenesis
EGCG: epigallocatechin gallate
ERR: estrogen-related receptor
FA: fatty acid
FAS: fatty acid synthase
FATP: fatty acid transport protein
FGF15: fibroblast growth factor 15
FOXO1: forkhead box protein O1
FXR: farnesoid X receptor
GLP1: glucagon-like peptide 1
GPAT: glycerol-3-phosphate acyltransferase
G6Pase: PGC1α-dependent enzyme
HDL: high-density lipoprotein
HFD: high-fat diet
HFrD: high-fructose diet
HL: hepatic lipase
HMGCR: 3-hydroxy-3-methylglutaryl coenzyme A reductase
HNF4α: hepatocyte nuclear receptor 4α
HSL: hormone-sensitive lipase
IDL: intermediate-density lipoprotein
IR: insulin resistance
LDL: low-density lipoprotein
LDL-c: LDL particles
LDLR: LDL receptor
LD: lipid droplet
LOX-1: lectin-like oxidized LDL receptor-1
LPL: lipoprotein lipase
LXR: liver X receptor
MAG: monoacylglycerol
MCD: MCD methionine and choline–deficient diet
MGL: monoacylglycerol lipase
MUFA: mono-unsaturated fatty acid
NADPH: nicotinamide adenine dinucleotide phosphate
NAFLD: non-alcoholic fatty liver disease
NE3: n-butanol extract of Panax notoginseng (Burkill) F.H. Chen
NPC1L1: Niemann–Pick C1-like 1
oxLDL: oxidized low-density lipoprotein
PCSK9: proprotein convertase subtilisin/kexin type 9
PGC1α: PPARγ co-activator 1α
PL: phospholipid
PNSs: Panax notoginseng (Burkill) F.H. Chen saponins
PPAR: peroxisome proliferator–activated receptor
PTE: pu-erh tea extract
SCD: stearoyl-CoA desaturase
SM: squalene monooxygenase
SR-BI: scavenger receptor class B type I
SREBP: sterol regulatory element–binding protein
T2D: type 2 diabetes
TAG: triacylglycerol
TC: total cholesterol
TCM: traditional Chinese medicine
TG: triglyceride
TrpV1: transient receptor potential cation channel subfamily V member 1
VLDL: very low–density lipoprotein
WHO: World Health Organization
WT: wild type
